# Merits and Pitfalls of Social Media as a Platform for Recruitment of Study Participants

**DOI:** 10.2196/47705

**Published:** 2023-10-11

**Authors:** Qutaibah Oudat, Tamilyn Bakas

**Affiliations:** 1 College of Nursing University of Cincinnati Cincinnati, OH United States

**Keywords:** recruitment, social media, review, study participant, methods

## Abstract

Efficient and effective methods of recruiting participants for studies have characteristically come with many challenges. The unprecedented rise of social media platforms such as Facebook and Instagram has revolutionized the ease of recruiting participants as compared to more traditional methods such as newspaper or radio advertisements. While these new advancements may seem to increase the success of recruitment, they are not without their own faults and limitations. In this paper, we intend to dissect the advantages and disadvantages of social media platforms in recruiting participants. Specifically, we will discuss the advantages of targeted and rapid recruitment, engagement, and cost reduction as well as the disadvantages of representativeness, privacy concerns, limited control, and limited access.

## Introduction

Historically, identifying, recruiting, and obtaining appropriate participants for scientific research studies, whether qualitative or quantitative, have been a challenging feat for several reasons [1,2]. One of the primary challenges is ensuring that the sample size is representative of the population being studied [3]. For example, if the study requires individuals with a particular health condition or personal characteristics, researchers may have difficulty finding eligible participants who meet specific demographic, geographic, or health criteria [4].

Over the past 2 decades, with the advent of social media, a gradual transition away from traditional methods and toward more digitalized and “soft” media sources, such as Facebook, Instagram, Twitter, Pinterest, and LinkedIn, has become more commonplace [3,5,6]. These methods have not only revolutionized previously insurmountable obstacles including visibility, accessibility, and participant diversity but have also affected aspects such as cost, time, and data collection tactics. In order to understand the ways in which social media has transformed modern study recruitment methods on a more intimate level, an objective comparison of social media and traditional methods is warranted. This review aims to investigate the advantages and disadvantages of using social media as a springboard for more efficient and effective recruitment of study participants. Construction of an accurate, comprehensive, and unbiased discussion about the current role of social media in scientific research will undeniably aid in bolstering understanding of its potential pitfalls and limitations, while also underscoring its unique attributes and strengths in this very dynamic field.

## Advantages of Using Social Media for Recruitment

### Overview

**Figure 1:**
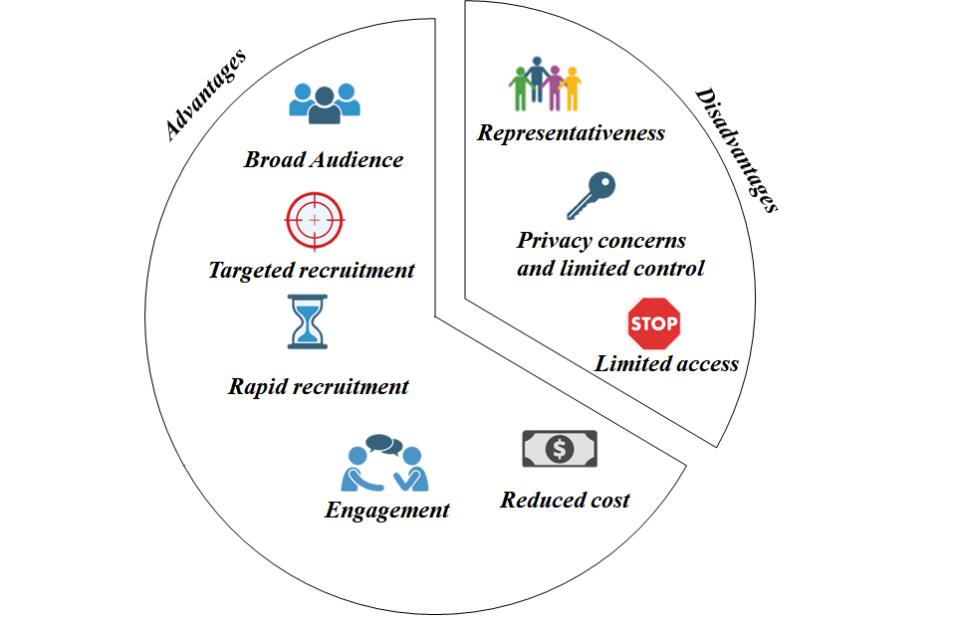
Advantages and disadvantages of using social media as a recruitment method.

Using social media as a recruitment platform, as illustrated in Figure 1, offers numerous benefits, including (1) broad audience, (2) targeted recruitment, (3) rapid recruitment, (4) engagement, and (5) reduced cost.

### Broad Audience

Although advertising through social media has the potential to reach millions of individuals worldwide, the use of social media for the recruitment of research participants is often overlooked [4]. Social media has the ability to reach a broad range of communities spanning a diverse array of geographical regions, cultures, languages, religions, ages, and socioeconomic status [7-9]. Traditional methods of participant recruitment (ie, without the use of social media) typically target specific recruitment sites in the community. These methods can result in selection bias and have limited the generalizability of countless studies. Selection bias is particularly detrimental for studies involving population health and epidemiology, as these studies are heavily reliant on accurate cohort representation [3].

### Targeted Recruitment

Social media possesses the ability to exclude or include different parameters and groups, allowing for optimal means to target one’s audience. Hence, researchers could simply target groups, clubs, specific demographics, and regions that were applicable to the study and hence increase the likelihood of finding participants who meet the study criteria. Traditional recruitment methods can be challenging, which dauntingly target the “public” audience, many of whom are not interested in the study nor eligible to apply for the study [10,11]. Moreover, traditional recruitment methods can result in considerable time, effort, and cost of screening potential participants for eligibility.

### Rapid Recruitment

Given the ease with which surveys can be generated, posted, and collected, social media platforms significantly decrease the time needed to recruit an appropriate cohort of participants. While much success depends on the participants’ willingness to click on the ad, sufficient interest, and willingness to follow through with the directions to enroll in the study, the ease of having an ad on one’s phone or laptop greatly facilitates the recruitment process and should not be underestimated. The fact that participants can enroll in a study from the comfort of their homes independent of time or day greatly increases the participant pool [6]. Traditional methods were notorious for taking more time than anticipated for both the participant and the study recruitment team, which has been shown in several studies to negatively impact the overall participation in the study [12].

### Engagement

Another attractive aspect of the use of social media platforms is that the advertisement can be constructed in such a way as to encourage engagement and even entertain potential study participants. Bright colors, fancy animations, charming sounds, and other tactics can be used to enhance the eagerness to read about the study and hence increase the likelihood for participants to join the study. This phenomenon is particularly interesting because traditional methods of recruitment tend to be very limited in this aspect. For example, newspaper or radio advertisements do not have this luxury and rely mainly on catchy phraseology or enthusiastic wording to grab the attention of participants, with little, if any, interaction from participants [13].

### Reduced Cost

Using social media has greatly reduced the cost of advertising as compared with older methods. Not only is there a decreased cost in constructing the advertisement but also in disseminating to and receiving feedback from participants. This reduced cost allows study coordinators to spend more funds on other aspects of the study, such as data analysis, participant compensation, and additional tools to aid in data collection. For example, the cost per participant on Facebook was shown to be less than traditional methods. A median value of US $14.41 for a typical Facebook advertisement fares well compared to US $1094.27 per participant for television recruitment, US $811.99 for printed media, US $635.92 for radio, and US $37.77 for email [3,14]. Carlini et al [15] had similar findings with a mean cost per participant of US $16.22 via Google ads, and between US $13.12 and US $250 for other traditional methods when recruiting young adults for weight gain analysis.

## Disadvantages of Using Social Media for Recruitment

### Overview

As we explore the multifaceted realm of using social media as a recruitment platform, it is essential to approach this subject with a comprehensive perspective. While the advantages are clear and noteworthy, it is equally crucial to recognize the potential challenges and drawbacks associated with this method of accessing potential participants (see Figure 1). The pitfalls of using social media as a recruitment platform can be summarized as (1) representativeness, (2) privacy concerns and limited control, and (3) limited access.

### Representativeness

Individuals who use social media actively tend to be younger in age, with the average age of a Facebook user being 38 years. Hence, studies geared toward pediatric, adolescent, or geriatric populations tend to not have sufficient representation on social media. Moreover, it is found that certain ethnic and racial groups tend to be more active on social media than others, again introducing significant selection bias to a study [16,17]. These factors taken together threaten the quality of data collection because the study population is strongly influenced by factors related to representation. Additionally, social media users may not be representative of the wider population, which can introduce bias into the sample and limit the generalizability of study results [18].

### Privacy Concerns and Limited Control

One of the major downfalls of using social media for the recruitment of study participants is the issue of breach of privacy. Several studies have indicated that many participants shy away from entering any personal information onto social media sites out of concern that their information may be leaked to third parties and misused [19]. This fear is a valid one, as several fraudulent sites obtain personal information such as bank account numbers and even social security numbers under the guise of being a study survey. Traditional methods of obtaining information from participants are less likely to encounter this problem and studies have shown that people were more trusting of traditional ways because they were associated with less duplicitous activity [20]. While researchers do their best to control privacy and target their respective groups of participants, social media is less controlled than the traditional methods. Surveys might be completed by individuals who do not meet the criteria, and verifying their identity is an onerous task, introducing a great degree of variability into a study, and in turn, endangering data quality [21]. Another essential point to discuss is the misrepresentation that exists with subject recruitment via social media, particularly when research subjects are being offered financial incentives to participate. This has become a very serious problem when recruiting from some platforms in particular, and it is increasingly common to see robots being used to complete surveys numerous times, demanding payment for each completion. However, many studies have overcome this issue by having participants click a box confirming that they are not robots, and much of the standard computerized software can detect this problem and inhibit the participant from proceeding.

### Limited Access

A prominent disadvantage of using social media is that not all individuals have access to such platforms. In the United States, more than 10% of the population does not have access to the internet either because of financial restrictions or because they consciously choose to live without it. Individuals and communities in this category may be excluded from important studies, simply for this reason alone [22]; this phenomenon would introduce a definite sampling bias to any study relying on social media. In contrast, more traditional methods, such as newspapers or radio, do not require internet and thus are arguably easier to access for this population [23-25].

## Conclusions

While social media recruitment has several advantages, researchers should carefully consider the potential limitations and assess whether it is the most appropriate recruitment method for their study. The choice of recruitment method will ultimately depend on the research question, the target population, and the available resources. Researchers should also consider combining social media recruitment with other methods to minimize potential bias and increase the representativeness of their sample.
